# Response and resistance to BRAF^V600E^ inhibition in gliomas: Roadblocks ahead?

**DOI:** 10.3389/fonc.2022.1074726

**Published:** 2023-01-09

**Authors:** Monica Capogiri, Andrea J. De Micheli, Alvaro Lassaletta, Denise P. Muñoz, Jean-Philippe Coppé, Sabine Mueller, Ana S. Guerreiro Stucklin

**Affiliations:** ^1^ Department of Oncology and Children’s Research Center, University Children’s Hospital of Zurich, Zurich, Switzerland; ^2^ Department of Pediatric Hematology and Oncology, Hospital Universitario Niño Jesús, Madrid, Spain; ^3^ Department of Medicine, Helen Diller Family Comprehensive Cancer Center, University of California, San Francisco, CA, United States; ^4^ Department of Neurology, Neurosurgery and Pediatrics, University of California, San Francisco, United States

**Keywords:** glioma, BRAF inhibitors, drug resistance, BRAF^V600E^ mutation, high-grade glioma (HGG), low-grade glioma (LGG)

## Abstract

BRAF^V600E^ represents the most common BRAF mutation in all human cancers. Among central nervous system (CNS) tumors, BRAF^V600E^ is mostly found in pediatric low-grade gliomas (pLGG, ~20%) and, less frequently, in pediatric high-grade gliomas (pHGG, 5-15%) and adult glioblastomas (GBM, ~5%). The integration of BRAF inhibitors (BRAFi) in the treatment of patients with gliomas brought a paradigm shift to clinical care. However, not all patients benefit from treatment due to intrinsic or acquired resistance to BRAF inhibition. Defining predictors of response, as well as developing strategies to prevent resistance to BRAFi and overcome post-BRAFi tumor progression/rebound growth are some of the main challenges at present in the field. In this review, we outline current achievements and limitations of BRAF inhibition in gliomas, with a special focus on potential mechanisms of resistance. We discuss future directions of targeted therapy for BRAF^V600E^ mutated gliomas, highlighting how insights into resistance to BRAFi could be leveraged to improve outcomes.

## 1 Background

Precision medicine brings new therapeutic avenues to clinical practice, tailoring therapies to tumor-specific vulnerabilities. Yet, with the increasing use of targeted agents, new patterns of tumor response and drug resistance are emerging. Tumor development under therapeutic stress is an evolutionary process. Treatment interventions impose selective pressures on tumor cells and their microenvironment that in turn impact response to therapy. A comprehensive understanding of a tumor’s biology and its evolution under treatment has thus become crucial for improving clinical outcomes, as well as successfully implementing novel cancer therapeutics.

Several targeted therapies are directed at activated kinases. BRAF^V600E^ is the most frequent mutation in BRAF (serine/threonine-protein kinase B-raf), a proto-oncogene and the most commonly mutated kinase in human cancers. The biological and clinical relevance of BRAF^V600E^ has been thoroughly described in melanoma, colorectal cancer, thyroid cancer, non-small-cell lung cancer, and hairy cell leukemia (1–4).

BRAF^V600E^ is also detected in glioma, the most common primary brain tumor across all ages (5, 6). Among gliomas, the highest incidence of BRAF^V600E^ is in low-grade gliomas, including pleomorphic xanthoastrocytoma (PXA, 56%), ganglioglioma (GG, 40%) and pilocytic astrocytoma (PA, 3%). While rarer in high-grade gliomas overall (< 5%), its incidence is higher in the epithelioid glioblastoma subtype (69%) (7).

Targeting BRAF^V600E^ with specific BRAF inhibitors (BRAFi) in monotherapy (8, 9) and in combination with downstream MEK inhibitors (MEKi) (10–12) led to improved patient outcomes in different cancer entities, including gliomas (13–17). Despite these encouraging results, patients may experience therapy failure at different stages of treatment, with some tumors being refractory to BRAFi upfront (intrinsic resistance), and others developing resistance, after an initial response, while on therapy (acquired resistance). Moreover, rapid tumor progression is often observed after BRAFi discontinuation (rebound growth).

Drug resistance is a multifaceted phenomenon involving genetic, epigenetic, metabolic and (micro)environmental changes (18, 19). Direct target reactivation, presence or gain of additional genetic alterations, activation of compensatory oncogenic pathways, and adaptive survival mechanisms are some of the features of cancer cells driving drug resistance. Additionally, environmental features such as hypoxia, blood-brain barrier (BBB) and tumor microenvironment (TME) may also play a critical role in response to treatment.

To improve outcomes and mitigate treatment failure, it is essential to understand, predict, prevent, and overcome resistance to BRAFi. In this review, we discuss the role of BRAF^V600E^ in glioma, with a focus on lessons learned from molecularly informed clinical trials as well as our current understanding of the mechanisms involved in resistance to targeted therapy. We highlight upcoming strategies to surpass potential roadblocks ahead.

## 2 Targeting BRAF

**Figure 1:**
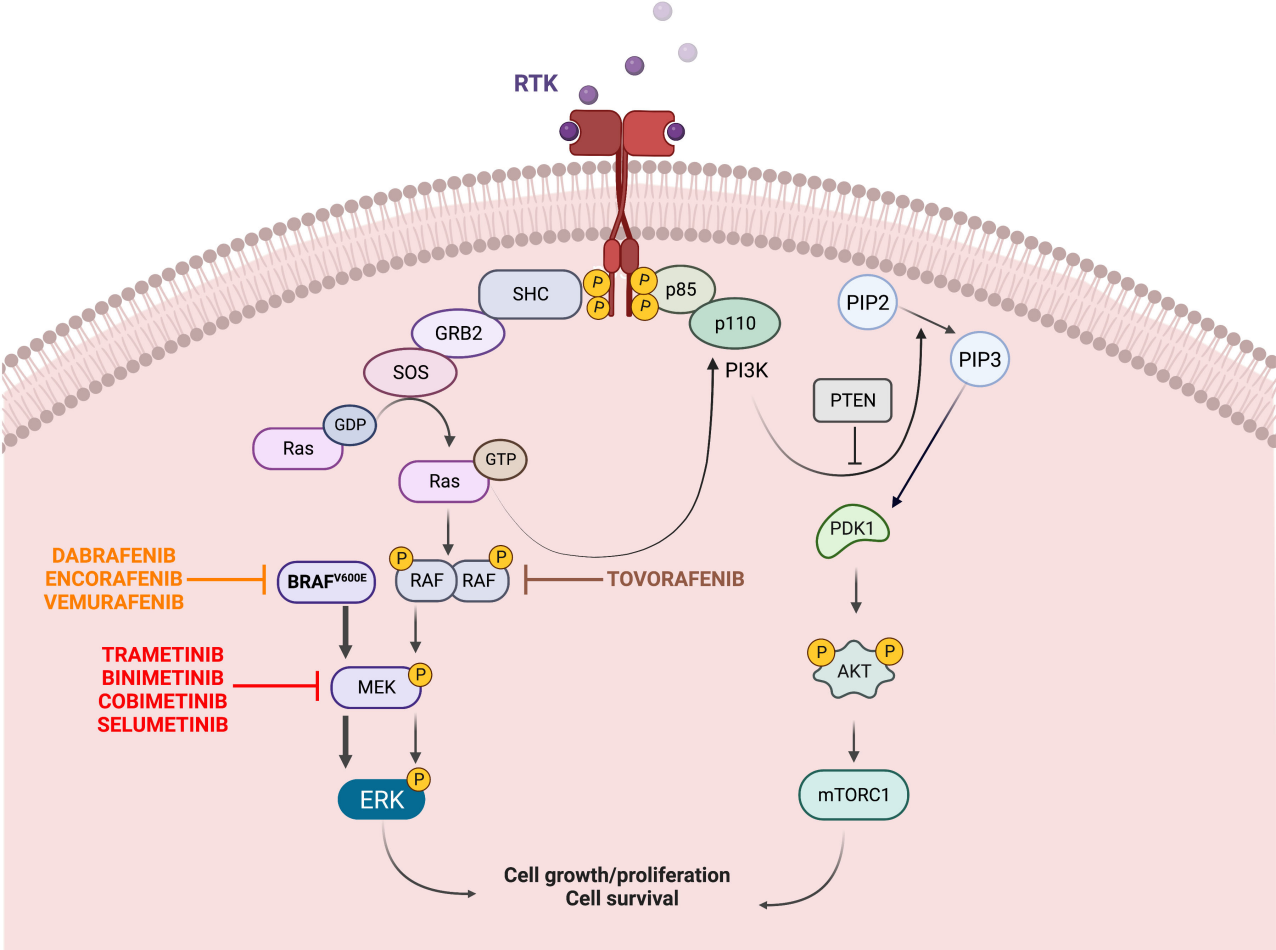
Oncogenic BRAF/MAPK signaling. The mitogen-activated protein kinase (MAPK) pathway is essential to the regulation of cellular growth, proliferation, and survival. Upstream of BRAF, growth factors binding to receptor tyrosine kinases (RTKs) at the cell surface lead to phosphorylation of RAS proteins, which then activate BRAF. Signal transduction continues downstream from BRAF to MAPK kinase (MEK) 1 and MEK2, and finally to ERK, which phosphorylates multiple targets, leading to increased cell survival, proliferation and differentiation. The V600E mutation results in constitutive activation of BRAF and downstream activation of MEK and ERK, in a RAS-independent manner. Specific inhibitors have been synthetized to target the mutated BRAF (Dabrafenib, Encorafenib, Vemurafenib) and the downstream MEK (Trametinib, Binimetinib, Cobimetinib, Selumetinib); more recently pan-raf inhibitors such as Tovorafenib have been developed. The PI3K/AKT/mTOR pathway is another survival pathway and its overactivation has been associated with BRAFi resistance (Created with BioRender.com).

The B-raf proto-oncogene (*BRAF*) is located on chromosome 7 (7q34) and encodes the BRAF protein of the RAF kinases family, a core component of the proliferation and survival RAS-RAF-MEK-MAPK cascade (**Figure 1**). In response to growth factor binding to receptor tyrosine kinases, RAF dimerizes and activates MEK1/2, which in turn phosphorylates ERK1/2. These events lead to the activation of multiple substrates that can promote cell growth and proliferation (20–22).

A missense mutation located in exon 15, nucleotide 1799, results in substitution of valine (V) by glutamic acid (E) at codon 600 (V600E) and causes a 500-fold increase in constitutive activity, without the need for dimerization (23). BRAF is mutated in nearly 7% of all human cancers, and V600E point-mutation represents more than 90% of observed alterations (24). In the same exon, other less frequent alterations have also been detected (V600K, V600D, V600R) (25).

The first generation of RAF inhibitors were small-molecule ATP-competitive multikinase inhibitors. Most notably Sorafenib (23, 26, 27), blocking CRAF, BRAF (wildtype and mutant), KIT, VEGFR1/2, FLT1 and PDGFR, was tested in several clinical trials. Unexpectedly, Sorafenib led to accelerated tumor growth in children with BRAF-fused and NF1-driven low-grade gliomas, most likely due to ERK/MAPK paradoxical activation (27). Selective BRAF inhibitors, includingVemurafenib (28–30), Dabrafenib (31, 32) and Encorafenib (33, 34), efficiently inhibit the catalytic activity (and MAPK signaling) of the mutant BRAF^V600E^, which can function as active monomers, but have limited efficacy against RAF in dimeric forms. Moreover, they can induce increase in functional RAF kinase dimers and paradoxical activation of MAPK signaling in wild-type BRAF or BRAF-fusion expressing cells (35).

A new generation of RAF inhibitors, developed after the characterization of BRAF dimerization, are now in clinical trials. Among these are “paradox breakers” (compounds such as PLX7904 and PLX8394) designed to avoid the paradoxical activation of MAPK. Another subclass includes compounds that inhibit both the monomeric and dimeric forms of BRAF, thus limiting the dimerization of RAF, a known mechanism of resistance (DAY101/Tovorafenib) (36).

## 3 Response to BRAFi: Monotherapy and combination with MEKi in gliomas

After several case studies (37–41), a growing number of clinical trials have established the clinical benefit of BRAF^V600E^-directed therapies in pediatric and adult gliomas (15, 16, 42). Reports of successful tumor responses and improved survival outcomes with favorable tolerability have been shown with Dabrafenib (42) and Vemurafenib (15, 17) as single agents or in combinations with MEK inhibitors (MEKi) (14, 16).

As part of the VE-BASKET multi-cohort study for non-melanoma cancers (15), BRAF inhibition with Vemurafenib led to an objective response rate (ORR) of 25% in a cohort of 24 adult patients with relapsed/progressive BRAF^V600E^ mutant gliomas. Reponses varied but were detected in all histological glioma subsets, with greater effect detected in low-grade gliomas and PXAs. Meaningful responses were also seen upon treatment with Dabrafenib in pediatric patients with low-grade gliomas [n=32, ORR 44% (95% CI 26 – 62)], with 1-year progression-free survival (PFS) of 85% (95% CI 64 – 94) (43). Supported by preclinical data showing a correlation of extent of MAPK inhibition with response to BRAFi treatment (44) and data emerging from trials evaluating MEK inhibitors (Selumetinib, Trametinib and Binimetinib) in pediatric glioma patients (45–49), subsequent studies assessed the effect of combinatorial BRAF^V600E^ and MEK inhibition.

Treatment with dabrafenib plus trametinib was evaluated in the phase 2 Rare Oncology Agnostic Research (ROAR) trial (16). An interim analysis showed an ORR of 69% (95% CI 39 – 91) in adult patients with low-grade gliomas (9 of a total of 13 patients). For the high-grade glioma cohort (n = 45, 31/45 with glioblastoma) a lower but meaningful ORR was reported of 33% (95% CI 39 – 91, 15 of 45 patients) at a median follow-up time of 12.7 months (IQR 5.4 – 32.3 months). In pediatric patients with low-grade gliomas, when compared to conventional chemotherapy with vincristine and carboplatin (VC), dabrafenib plus trametinib significantly increased ORR (47%, 95% CI 35-59 *vs* 11%, 95% CI 3-25 for the VC cohort; odds ratio 7.2) (14). Comparison with previous single-agent trials is limited by differences in design and heterogeneity in patient inclusion, however current data suggest an advantage of combinatorial BRAF and MEK inhibition, when compared to BRAFi monotherapy. Beyond progression-free and overall survival, time to response is another important aspect of therapy. Rapid tumor shrinkage is especially relevant in CNS locations where tumors can lead to potentially irreversible neurological damage (e.g. patients with optic pathway gliomas, for whom preservation of vision is critical). Future studies should provide further insights regarding extent/timing of response using targeted treatments, compared to conventional chemotherapy.

The therapeutic benefit of BRAFi and concurrent BRAFi/MEKi in gliomas is indisputable (14, 16). Whether complete and sustained tumor responses can be achieved remains unclear and several key questions remain to be addressed. A large subset of patients may experience rebound growth upon discontinuation of treatment (13). However, most data so far were collected for patients with progressive gliomas, treated with BRAFi after one or several lines of conventional cytotoxic therapies. The optimal timing for introduction of targeted agents (upfront *vs* at progression), duration of treatment, schedule for treatment discontinuation and best approach to post-BRAFi progression remain under debate. Also, long-term side effects of these targeted therapies in children remain unknown.

## 4 Resistance to BRAF inhibition

The tumor’s ability to escape therapeutic pressure can precede (intrinsic resistance) or emerge during treatment (acquired resistance). As a result, patients can be refractory to treatment or experience an initial response followed by tumor growth while on treatment. Tumor progression after BRAFi discontinuation (rebound growth) is another form of treatment failure but not strictly driven by resistance to BRAFi, since it occurs also in patients with good response to treatment, in the absence of intrinsic or acquired resistance.

**Figure 2:**
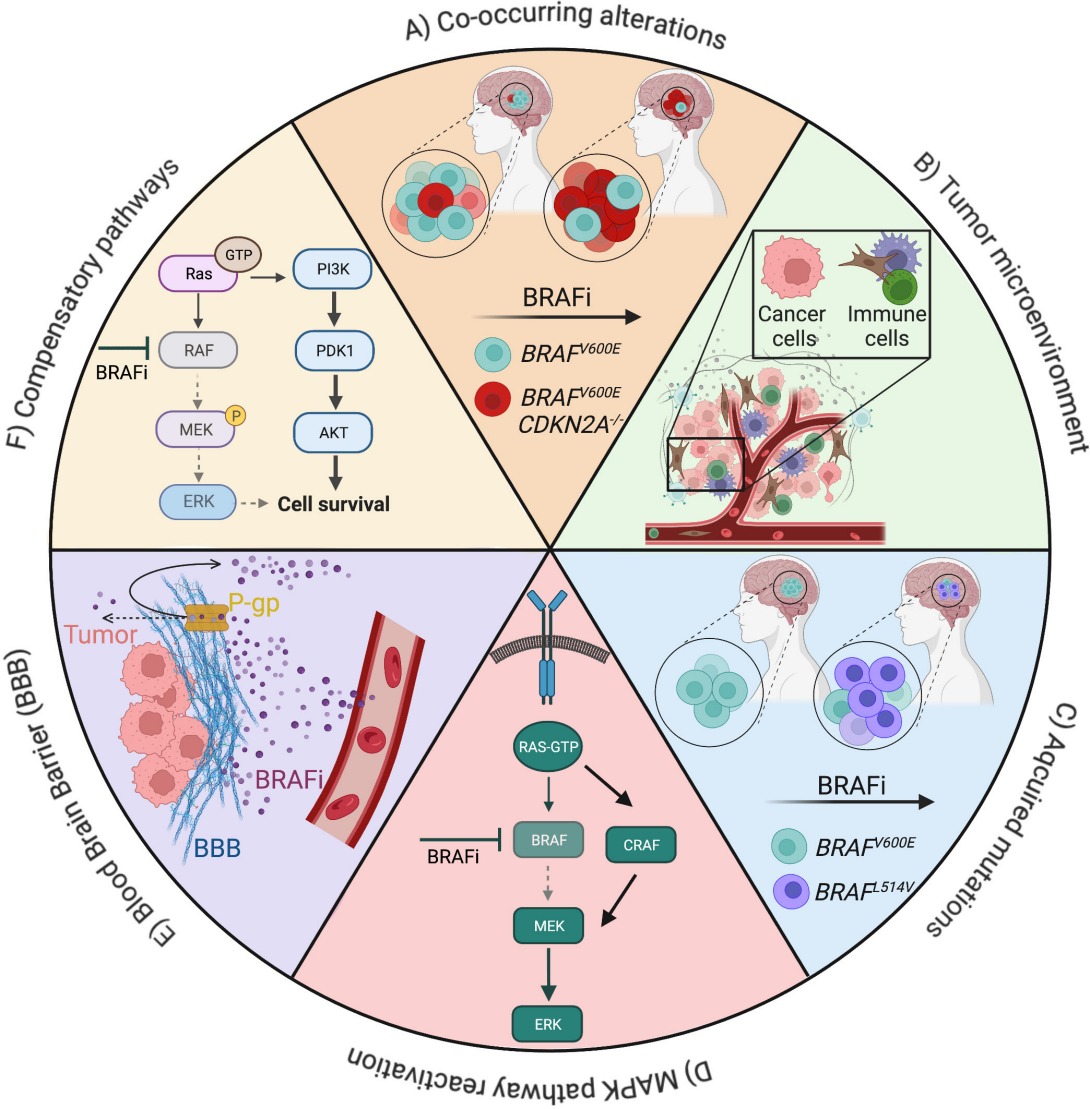
Overview of mechanisms of resistance to BRAF inhibitors (BRAFi) in gliomas. Drug resistance can be driven by cell-intrinsic factors, such as mutations **(A, C)**, pathway activation **(D, F)**, cell-extrinsic factors, such as the tumor microenvironment **(B)**, the presence of physical barriers like the blood brain barrier and its P-glycoprotein **(E)**. Examples of alterations and signaling pathways related to resistance to BRAFis in low- and high-grade gliomas are depicted (Created with BioRender.com).

In a general context, intrinsic resistance to targeted therapy is usually caused by pre-existing concomitant genetic alterations. Resistance may also derive from a non-genetic rewiring of signaling pathways, epigenetic modulation and/or changes in the tumor microenvironment, leading to the activation of compensatory signals and/or reactivation of the targeted pathway (50–52) (**Figure 2**).

### 4.1 Concomitant alterations

A common reason for BRAFi treatment failure is the presence of concomitant response-modifying alterations (53, 54). *CDKN2A* deletion can co-occur with BRAF^V600E^ in different cancer entities, including gliomas, where it is associated with a higher risk of progression, malignant transformation, and overall poorer outcome (55, 56). Intrinsic resistance to BRAFi has been associated with PI3K-mTOR pathway aberrations in several BRAF^V600E^ mutated non-melanoma tumors, including glioblastoma (57). All patients presenting PI3K-mTOR pathway mutations (such as PTEN^P339fs* 2^, PIK3CA^I391M^, AKT^D46E^) and BRAF^V600E^ progressed within less than 3 months of targeted monotherapy. Later clinical trials further corroborated this observation: five pediatric and young adult patients with BRAF^V600E^-mutated brain tumors and PI3K pathway alterations (58) were treated with a combination of Vemurafenib and Everolimus. Overall, two patients (40%) had a partial response and one (20%) had stable disease as best response. Similarly, H3.3 K27M mutation is a poor prognostic marker in gliomas overall and has been detected with concomitant BRAF^V600E^ mutation in rare cases (13, 59).

BRAF^V600E^ has been detected in a tumor harboring also a BRAF-KIAA1549 fusion. In a phase 1 trial of the MEK inhibitor Selumetinib (45), 1 out of 38 pLGG patients carried both alterations, and rapidly progressed on treatment. Though thought to be generally mutually exclusive, it is unclear how patients with BRAF^V600E^ gliomas harboring additional genetic alterations at the level of BRAF (mutations and/or fusions) respond to BRAFi.

### 4.2 MAPK pathway reactivation

About 80% of drug resistant cases in BRAF^V600E^ mutated tumors overall are thought to arise from the reactivation of MAPK pathway (60). Despite positive initial response to inhibitors such as Dabrafenib or Vemurafenib, it is well documented that cells can find alternative ways to activate the pro-survival MAPK pathway in many cancer subtypes (61, 62), including gliomas (63). Secondary mutations in the MAPK pathway, *BRAF* copy number gains, BRAF alternative splicing as well as increased expression of receptor tyrosine kinases are some of the diverse mechanisms that can cause MAPK pathway reactivation (64).

Binding of BRAFi to one monomer induces allosteric transactivation of the second monomer, while at the same time the binding of the drug at the second monomer is sterically prevented (65). In tumor cells bearing BRAF^V600E^ mutants, the mutated form can dimerize with BRAF^V600E^, wild-type BRAF and wild-type CRAF, leading to MAPK pathway reactivation (35).

Mutations or hyperactivation in the PI3K/AKT pathway can also cause the reactivation of MAPK (66). In patient-derived BRAF^V600E^ HGG xenograft models chronic exposure to Dabrafenib led to an up-regulation of RAS, phospho-c-Raf, phospho-p90RSK, and phospho-AKT, with paradoxical activation of the MAPK pathway and AKT/mTOR pathway causing resistance to BRAFi (67).

### 4.3 Activation of compensatory oncogenic pathways

Signaling redundancies and interconnections through pathway crosstalk have been identified as contributors to drug resistance (68). Negative feedback loops, cross inhibition, cross activation, and pathway convergence are hallmarks of cell signaling regulation. Activation of compensatory pathways is more likely to occur in HGG when compared to LGG, typically driven by a single alteration resulting in up-regulation of the RAS/MAPK pathway (69, 70).

Feedback activation of EGFR signaling is one way by which BRAF^V600E^ gliomas adjust and escape BRAFi treatment. BRAF^V600E^ inhibition can suppress MAPK signaling, which in turn downregulates the EGFR phosphatase PTPN9, resulting in sustained EGFR phosphorylation and enhanced EGFR activity (71). One case report supports this observation, in which a glioblastoma patient carrying both BRAF^V600E^ and an activating mutation of *EGFR* experienced no clinical benefit from RAF and MEK inhibition (72).

Additional pathways have been implicated in resistance to BRAFi. For example, Vemurafenib-resistant glioma cells up-regulate pro-survival mediators such as Wnt and increase Axl receptor tyrosine kinase activity (73).

### 4.4 Acquired mutations

In melanoma, around 50% of patients treated with BRAFi alone or in combination with MEKi experience an initial significant shrinking of the tumor followed by tumor re-growth due to the acquisition of a new mutation (74). Loss-of-function mutations in *STAG2* (75), *BOP1* downregulation (76) and *COT* alteration (77) are just some of the pro-survival mutations that bypass the inhibition of MAPK pathway in melanoma. A case report described an acquired BRAF^L514V^ mutation at time of relapse in a BRAF^V600E^ pLGG, leading to dimerization of BRAF^V600E^ and rendering the monomer-specific inhibitor inefficient (78). In a larger case series, paired pre- and post-BRAFi pediatric and adult glioma samples (n = 15 patients) were analyzed, and 9 cases displayed a novel mutation acquired after BRAFi monotherapy or in combination with MEKi (79). Among these alterations, loss of *NF1*, *PTEN*, *CBL* and *CRAF* activation were functionally validated using patient-derived cell lines. Further alterations included mutations in *PIK3C2G*, *ERRFI1*, *BAP1*, *ANKHD1*, and *MAP2K1*. This study also revealed the heterogeneity of mechanisms of resistance to BRAFi, with all post-treatment samples with acquired mutations presenting unique novel alterations.

### 4.5 Blood-brain barrier and challenges in drug delivery

The BBB and blood-tumor barrier constitute two sequential barriers that drugs need to cross to reach the tumor. Most commercially available drugs have a reduced capability of crossing the BBB, thus representing an important limitation to therapy effectiveness in brain tumors. The presence of tight junctions and efflux proteins on the BBB impacts distribution of BRAFi and MEKi within the central nervous system. P-glycoprotein (P-gp) and breast cancer resistance protein (BCRP), expressed on the luminal side of the BBB, can actively efflux various therapeutic agents (80–82) including Vemurafenib, Dabrafenib, Trametinib, and Cobimetinib (83, 84). Furthermore, many kinase inhibitors such as Dasatinib, Gefitinib, Sorafenib and Erlotinib (85–87) are substrates for important transporters at the BBB and this significantly limits their concentrations in the brain.

Although it is not clear whether these findings have a direct link with therapy failure in glioma patients, it is likely that intrinsic resistance to targeted therapy is partially due to the inability of some drugs to reach therapeutic concentrations in the brain.

## 5 TME and immune system

Like the BBB, the tumor microenvironment (TME) can impose additional roadblocks to targeted therapies. While most studies have been performed on melanoma, some of the conclusions could be applicable to gliomas. The TME contains various non-cancerous cells such as astrocytes, endothelial cells, microglia, and macrophages, which by their own distinct mechanisms can limit the efficacy of targeted inhibitors. For example, TME stromal cells secrete high levels of ligands capable of activating the MAPK pathway through receptor tyrosine kinases, thereby activating the same pathways that BRAFi are designed to block (83). In BRAF^V600E^-mutant melanoma, a positive correlation between intrinsic resistance to BRAFi and HGF expression by stromal cells has been described (88).

BRAFi also affects the immune phenotype of the TME in ways it can either benefit treatment or explain mechanisms of resistance. For instance, BRAFi have been associated with an increased number of T-cells in the TME and up-regulation of MHC proteins on cancer cells (89, 90). In a study on metastatic melanoma, patients treated with Dabrafenib and Trametinib displayed an increase in T-cell infiltration and an increase in melanocytic differentiation agents (91). While these changes in the immune composition could facilitate BRAFi treatment, other changes observed in the TME were also associated with resistance. For examples, BRAF inhibition have also been linked to an increase in expression of the immunosuppressive ligand PD-L1 (91). While the mechanisms by which the immune phenotype of the TME evolves to adapt to treatment are yet to be fully characterized, these studies suggest that better therapeutic benefit could be derived from combining BRAFi with immune checkpoint inhibitors or other immunomodulating drugs.

## 6 Future perspectives

BRAF inhibitors have shown promising results against a wide variety of BRAF^V600E^ mutated cancers. For children,adolescents, and adults with low-grade gliomas, responses to BRAFi are superior to conventional chemotherapy and a growing body of evidence supports upfront targeted therapy in these patients. Though to a lesser extent, encouraging responses further support the use of BRAFi in the treatment of pediatric and adult patients with HGG.

With BRAFi now part of routine clinical care, new challenges emerge. The most pressing concern at present is resistance to targeted therapy as a cause of treatment failure, morbidity, and mortality. In many human cancers, important progress has been made using genomic and transcriptomic profiling of paired clinical samples to track the tumor evolution during and after targeted treatment (92, 93). Yet, re-biopsy at time of relapse is not a standard of care in patients with relapsed gliomas and data are limited.

Several ongoing preclinical and clinical efforts are expected to further elucidate the molecular causes underlying BRAFi treatment failure. Recent reports highlight the evolution of gliomas under BRAF inhibition and reveal a high diversity of acquired alterations at relapse (79). This heterogeneity also suggests that the mechanisms driving drug resistance are patient-/tumor-specific, further supporting the need for tumor biopsies at time of relapse. Though challenging, such an approach may reveal tumor-specific vulnerabilities as well as inform the design of new drug interventions and personalized treatment options for patients with otherwise limited treatment options.

One of the main mechanisms of resistance to BRAFi in gliomas is reprogramming of kinase and cell signaling activity. Several proteomic approaches such as Reverse-Phase Protein Array (RPPA), Mass Spectrometry (MS; total or phospho), or Inhibitor-Bead Mass Spectrometry (IB-MS) can reveal how tumors rewire signaling cascades upon therapeutic pressure but require relatively large amounts of sample. Another recently developed method is the High Throughput-Kinase Activity Mapping (HT-KAM), which measures the activity of hundreds of kinases simultaneously, in cells or in tumor tissues’ extracts (94, 95). The readout of this assay provides a rank of kinases that are overactive/underactive upon treatment, thus uncovering parallel/orthogonal mechanisms that can be targeted to overcome therapeutic resistance. With such understanding of the molecular mechanisms treatment failure, BRAFi could then be rationally combined with inhibitors of resistance mechanisms, to increase treatment efficacy.

Lastly, very little is known about the long-term side effects of these therapies, especially in pediatric patients (96). Their potential impact in the development of young children and adolescents must be carefully assessed in comprehensive longitudinal prospective studies. Advanced imaging- and/or liquid biopsy-based strategies for disease monitoring are further areas of research, expected to improve how we predict response, monitor and tailor treatment with BRAFi. Translation of new technologies and innovation into clinical care will thus be key to understand and overcome BRAFi resistance.

## Author contributions

MC, AD and AS wrote the first draft of the manuscript. MC generated the figures. All authors contributed to writing and editing of the final manuscript. All authors listed in the paper have made a substantial, direct, and intellectual contribution to the work and approved it for publication. All authors contributed to the article and approved the submitted version.
